# An Overview of Artificial Intelligence Applications in Radiological Imaging for Bone Fracture Diagnosis

**DOI:** 10.7759/cureus.97065

**Published:** 2025-11-17

**Authors:** Jina Shargawi

**Affiliations:** 1 Psychiatry, Ealing Hospital, London, GBR

**Keywords:** artificial intelligence, convolutional neural networks, deep neural networks, fracture detection, radiology

## Abstract

Fractures are becoming a significant health concern, especially in the aging population, making early detection and accurate management essential for optimal patient outcomes. Standard radiographic imaging methods are the primary diagnostic tool for fractures; however, subtle or complex fractures are often overlooked by radiologists, resulting in delayed treatment and an increased risk of complications. Adding artificial intelligence (AI) to radiology would make diagnoses more accurate, reduce wait times, and make it easier for doctors to make decisions in busy, urgent situations.

This literature review discusses contemporary evidence regarding the role of AI in radiology and fracture detection, emphasizing machine learning models, particularly convolutional neural networks (CNNs) and deep neural networks (DNNs). It also explores critical aspects such as clinical workflow, diagnostic efficacy, and the ethical dilemmas associated with the integration of AI technologies in healthcare.

The results show that AI systems are highly sensitive in detecting subtle and complex fractures and, in some cases, perform better than radiologists. AI algorithms can also help doctors prioritize urgent cases, speed up reporting, and lower the risk of missing fractures in busy settings like the emergency department. Nonetheless, further research is required before integrating AI into the healthcare system, as challenges often overlooked remain, including data privacy concerns, algorithmic bias, and the need for radiologist oversight to ensure patient safety. This literature also discusses ethical issues, such as how to maintain clinicians' autonomy when using AI as a decision-support tool.

In general, using AI in radiology is beneficial when clinicians use it as an additional tool to detect fractures. It can help make diagnoses more accurate, speed up the process, and improve patient outcomes. However, future research should focus on extensive and practical studies, develop strategies to ensure the safe and ethical incorporation of AI into clinical practice, and enhance radiology training in AI. This review highlights the potential benefits of AI in healthcare while emphasizing the need to address the ethical dilemmas and challenges that arise.

## Introduction and background

Fractures are increasingly becoming a primary health concern worldwide, placing significant demands on healthcare systems [1]. They are among the common injuries that not only cause chronic pain but also lead to long-term problems such as loss of mobility and independence [1]. Experiencing fractures is estimated to affect one in three women and one in five men over the age of 50, making it a prevalent health issue [2]. A study has shown that the number of cases will rise by almost 70% in the coming years due to aging populations [3]. The increase in fracture incidence makes fractures not just an individual problem but a growing public health burden. In England and elsewhere, healthcare systems are already under pressure from the costs of hospital admissions, rehabilitation, and ongoing care linked to fracture injuries [4].

Radiology has always played a significant role in fracture diagnosis. The management and treatment of fractures rely heavily on imaging, from the earliest X-rays in the late 1800s to more advanced technologies such as computed tomography (CT) and magnetic resonance imaging (MRI) [5]. CT, for instance, shows bones in three dimensions, which makes it easier to detect minor and complex fractures [6]. Nevertheless, even with these improvements, detecting fractures remains difficult. Studies have shown that a significant proportion of fractures are missed; one report found that nearly 13% of lumbar fractures were missed in trauma patients treated in emergency departments [7]. There are multiple reasons for this, including busy emergency departments, high-pressure environments, and heavy workloads on radiologists, which can lead to fatigue [8]. In addition, some fractures are very small or hard to detect. Reporting delays are also becoming a growing problem because there are not enough radiologists, and more people need imaging as they get older [8].

Rising imaging demand due to aging populations and the broader use of cross-sectional imaging has stressed services, and artificial intelligence (AI) is emerging as a tool to help manage workload. The National Institute for Health and Care Excellence (NICE) has recognized the potential of AI and supports its use in fracture detection, assisting clinicians in managing the growing workload [9]. AI systems, especially those that use machine learning and deep learning, have shown the ability to identify small patterns in images that the human eye often misses [10]. For instance, convolutional neural networks (CNNs) and deep neural networks (DNNs) are artificial neural networks that can learn from thousands of images and detect subtle details indicative of fractures [10]. AI can help radiologists identify abnormal scans, speeding up reporting and ensuring that patients receive timely treatment. In emergency situations, this could be life-changing by enabling faster decision-making and reducing wait times [11].

Although AI offers multiple benefits for improving the accuracy of fracture detection and treatment, significant research gaps remain obstacles [12]. Clinical translation is considered one of the major research gaps that need further exploration [13]. A major limitation of current literature is that most studies evaluate AI models in controlled research settings rather than in real-world clinical practice, which affects their overall performance [14]. In addition, several ethical issues arise from the integration of AI into healthcare. Algorithmic bias is one of these issues, given that AI can be trained on small datasets, leading to less accurate results for certain populations [15]. This not only raises ethical concerns but can also limit the safe and effective clinical use of these systems. Furthermore, the decision-making processes of AI models are frequently ambiguous and opaque, a characteristic commonly described as the "black box" phenomenon, which requires further investigation to address ethical concerns [16]. Questions about professional independence also arise: how much should radiologists depend on AI, and who is responsible if an error occurs? These doubts and ethical issues highlight the importance of conducting thorough research and carefully integrating AI into radiology [17].

If AI is carefully integrated into and used within the healthcare system, it could serve as a powerful tool to assist radiologists rather than replace them. It might reduce the number of missed fractures, shorten delays and waiting times, and ultimately improve patient outcomes [11]. It can also help radiologists with decision-making, prioritizing urgent cases, and reducing workload, particularly in emergency departments [11]. However, if AI developers ignore existing research gaps, it could reduce trust in healthcare and put patients at greater risk of harm [18]. This paper presents a narrative literature review of peer-reviewed studies published up to 2025, aiming to investigate the contemporary applications of AI in radiology for fracture diagnosis and the obstacles that AI developers must address to ensure the safe and effective incorporation of AI into clinical practice.

## Review

Artificial intelligence and deep learning in medical imaging

AI is an umbrella term that encompasses deep learning (DL), machine learning (ML), and neural networks. ML and DL both contribute to performing intellectual tasks similar to those carried out by the human brain [19]. However, ML involves creating a computer model that can learn and make predictions based on the provided data [20]. DL, on the other hand, is considered a subset of ML and is based on neural network structures that mimic the human brain. These structures can automatically learn discriminative features from datasets [21]. Based on the number of layers, we can distinguish between artificial neural networks (ANNs) and deep neural networks (DNNs). An ANN has only one hidden layer, whereas a DNN has multiple hidden layers, which allows it to learn more complex data and make fewer errors [20].

Convolutional neural networks in fracture detection

**Figure 1:**
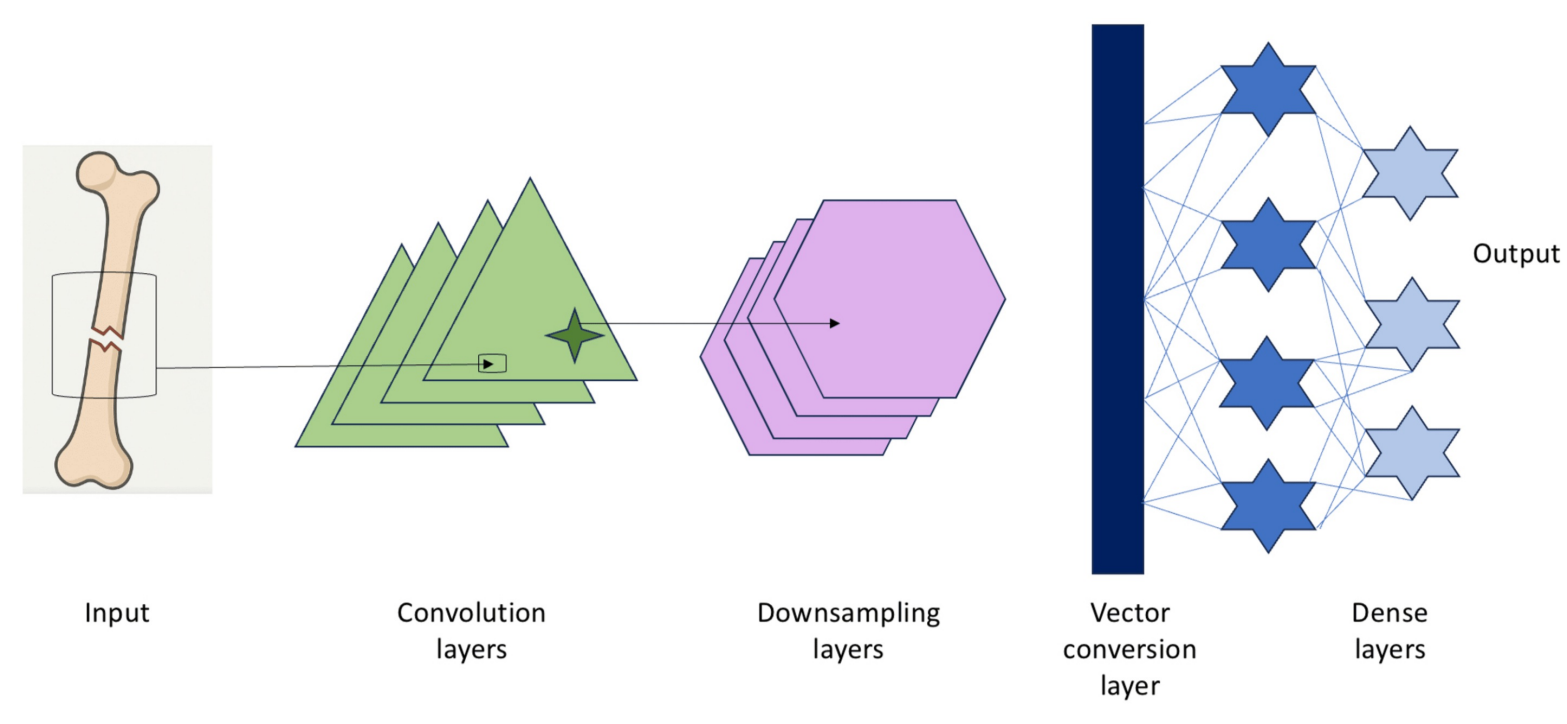
Example of a general architecture of a Convolutional Neural Network (CNN). Figure created by the author to illustrate the general architecture of a convolutional neural network (CNN), based on standard models described in Sarvamangala et al. [22].

CNNs are a type of DNN that use filters to extract features from images [20]. Their primary function is to learn patterns and combine them to recognize more complex images [22]. Radiologists are currently using CNNs in the analysis of medical imaging; for example, they assist in detecting scaphoid fractures on plain X-rays [20]. Figure 1 illustrates the steps by which CNNs detect fractures and generate a diagnosis. CNNs consist of neurons that recognize patterns in images, with convolutional layers identifying basic features such as edges, shapes, and bone textures [22]. The activation function then helps the model focus on the key parts of the image and highlight the fracture in the bone. After identifying the fracture, the pooling step reduces the image size while retaining key abnormal details to pinpoint the fracture location [22]. The final step involves fully connected layers, which gather all information and formulate a diagnosis [23].

CNNs and DNNs are highly effective at recognizing subtle fractures that radiologists may miss; however, the black-box nature of these models should not be overlooked [16]. There is no comprehensive understanding of how an AI algorithm arrives at its final diagnosis, although the input and output of these models are known. There is a lack of transparency in the intermediate steps and the way AI models combine features [16]. This lack of interpretability and the hidden internal decision-making processes are why these models are often referred to as "black boxes" [16]. Research has shown that advancements could help address this issue. For instance, one study used gradient-weighted class activation mapping (Grad-CAM) in its AI model to show which parts of the X-ray the system examined when detecting scaphoid fractures [24]. This AI model enhances transparency and provides valuable insights into how it makes decisions.

Clinical performance and diagnostic accuracy

Researchers have found that CNNs have significant potential to improve fracture detection in CT trauma scans [25]. Deep-learning models can identify complex fractures that clinicians often miss. These models recognize patterns on CT scans that could indicate the presence of a fracture [26]. In a trauma setting where time is critical, CNNs can provide accurate diagnoses efficiently, resulting in faster treatment planning and improved patient outcomes [27]. CNNs provide a decision-support tool that minimizes the potential risk of missed fractures, especially when fractures are non-displaced or subtle. A retrospective study of 4,480 radiographs from a single-center trauma cohort showed that CNNs, when combined with clinical reports, reduced missed fractures by 88% [25]. However, an important point to remember is the number of datasets required for training CNN models; smaller datasets can lead to overfitting and reduced accuracy.

Another common injury of the carpal bones is a scaphoid fracture, which occurs in 82-89% of cases, most commonly in young adults with wrist trauma [28]. Recognizing and treating the condition early can help reduce the risk of complications such as osteoarthritis and avascular necrosis [28]. X-rays are the most common modality used to detect scaphoid fractures because they involve minimal radiation, are affordable, and are readily accessible. However, X-rays have low sensitivity for diagnosing these fracture types [28]. MRI and CT scans are considered more sensitive for detecting occult scaphoid fractures; however, CT involves higher radiation exposure, and MRI is associated with higher costs [24]. Therefore, AI developers can utilize CNNs to help detect scaphoid fractures on X-rays, maintaining both clinical and economic effectiveness. A model was developed consisting of two CNNs that detect scaphoid segmentation and fractures. Lee et al. compared this model with a radiologist's interpretation of scaphoid fractures and found that the CNN model produced better outcomes [29]. However, study designs employ different methodologies and patient populations, which can lead to discrepancies in results and complicate the generalizability of findings. In addition, inconsistent training of AI models leads to a lack of standardization in practice [28]. These are important aspects that research needs to address to improve the application of AI in fracture detection.

Radiologists can use AI as a supportive tool to aid in decision-making. Studies indicate that fracture detection improves significantly when radiologists use AI as a decision-supporting tool, particularly in emergency situations [30]. In a retrospective multi-reader study of 500 consecutive trauma patients (232 women, 268 men; mean age 37 ± 28 years, range 0.25-99 years), AI-assisted radiologists increased the patient-wise sensitivity of fracture diagnosis by up to 20% [31]. Another retrospective multi-reader study involving 15 readers assessing 340 appendicular skeleton radiographs reported a 29% reduction in missed fractures when AI support was used [30]. These improvements are primarily due to AI enhancing radiologists' confidence and enabling faster, more accurate diagnoses. Additionally, AI accelerates decision-making and improves workflow efficiency in busy clinical settings by reducing the time required to interpret scans. Thus, a key benefit of AI in fracture detection is its ability to increase sensitivity, shorten reporting times, and ultimately provide smoother workflows and improved patient outcomes [32].

Ethical and professional challenges

AI has the potential to make diagnoses more accurate, streamline workflows, and improve patient care, which is why the field of radiology is changing rapidly. However, the integration of AI into clinical practice poses numerous complex challenges that require appropriate resolution, including ethical, legal, and professional issues [12]. Algorithmic bias, for instance, can arise when datasets used to train AI systems are not diverse, leading to differences in care across patient populations [15]. Similarly, heavy reliance on AI for diagnosis can affect professional autonomy, raising concerns about overdependence [33]. The protection of patient data is another significant issue, as machine learning requires access to large volumes of medical records, thereby increasing the risk of privacy breaches [12]. These challenges are often overlooked and must be addressed promptly to protect clinical autonomy, patient privacy, and public trust in the healthcare system [15].

Algorithmic bias is one of the major ethical dilemmas that requires further research. Developers train AI systems on pre-existing data that typically come from a single population; therefore, their performance can be significantly reduced when applied to patients from other backgrounds, introducing biases [15]. In addition, there is a lack of transparency in how AI processes data, making it difficult to detect and correct such biases. These ethical dilemmas raise concerns regarding accountability and can lead to treatment errors and missed diagnoses [33]. Furthermore, patients have expressed moderate skepticism about AI's ability to manage their care and voiced concerns regarding the potential for medical errors [18]. Addressing algorithmic bias requires an approach that includes using diverse training datasets, developing explainable AI models, and continuously monitoring performance [15]. By managing and addressing these ethical risks, healthcare systems can use AI to improve patient outcomes while maintaining diagnostic accuracy and patient confidence [15].

Professional autonomy is another significant ethical challenge when integrating AI into radiology. While AI can enhance decision-making and support radiologists in fulfilling their ethical responsibilities [34], radiologists should use AI in clinical settings as a supplementary tool rather than a replacement. Relying heavily on AI could make doctors less knowledgeable and reduce their ability to make independent clinical decisions [34]. Physicians may continue to use AI to assist in interpreting scans and making treatment decisions, as long as they remain autonomous [17]. Protecting professional autonomy fosters accountability, as physicians maintain responsibility for patient outcomes and sustain patients' trust in the healthcare system [17]. Therefore, to ensure that AI is used ethically in healthcare, it is essential to strike a balance between AI assistance and clinical judgment. Hospitals can achieve this by implementing local policies that require radiologists to review AI-generated findings before finalizing diagnoses, ensuring that AI supports rather than replaces clinical decision-making.

Legal implications and data governance

Policymakers should also consider how to protect patient data to maintain confidentiality. AI systems require large datasets; however, AI developers must handle data appropriately to preserve privacy and prevent misuse [35]. Regulatory bodies should establish transparent governance to ensure the proper collection, storage, and use of patient data, thereby promoting patient safety and trust. To maintain transparency, patients should always be informed about how developers use their data for AI training and who has access to it [35]. Ongena et al. conducted a questionnaire that demonstrated patients prefer to be actively informed and aware of how clinicians use AI systems in their care. Therefore, to implement AI effectively in radiology, the healthcare system must carefully consider social factors such as trust, communication, and patient engagement to avoid undermining public confidence in healthcare [18].

Education, training, and the future of AI in radiology

Despite the rapid development of AI applications in radiology, a gap remains in existing studies. Most of the literature focuses mainly on the technical performance of AI models, such as accuracy and sensitivity in detecting fractures, without addressing the uncertainties and research gaps that accompany them [36]. Several areas require further investigation and implementation to improve outcomes in the future. These include the clinical translation of AI in radiology and the legal issues associated with its use [13]. To address these challenges, careful governance, stakeholder engagement, and dedicated education are essential [15]. By closing these gaps, AI has the potential to improve diagnostic accuracy, workflow efficiency, and patient care. Table 1 summarizes the key research gaps in AI applications in radiology, the challenges they pose, and the potential future measures required to ensure safe and effective implementation.

**Table 1:** Applications of artificial intelligence in radiology: research gaps and future directions Evidence levels were assigned according to the study design. Level 1 corresponds to systematic reviews or meta-analyses of randomized controlled trials. Level 2 includes individual randomized controlled trials. Level 3 covers observational analytic studies, such as cohort, case-control, or cross-sectional studies. Level 4 represents case series or poor-quality cohort studies, and Level 5 corresponds to expert opinion or narrative review articles.

Research Gap	Current Issue	Future Direction	Reference	Evidence Level
Clinical translation	Good performance in labs, but not in real-world settings	More real-life trials in diverse populations	Panayides et al., 2020 [14]	Level 5
Legal liability	Unclear who is responsible for AI errors	Clearer regulations and accountability frameworks	Aldhafeeri et al., 2024 [15]	Level 3
Education	Radiologists lack AI training	Integrate radiology into AI curricula	Tejani et al., 2023 [37]	Level 5

Clinical translation is one of the most significant gaps in research and an important barrier to the use of AI in radiology [38]. Many AI tools perform well in controlled research settings but do not always demonstrate the same effectiveness in real-world clinical environments [14]. AI developers need a structured approach that includes identifying a specific clinical problem for AI to solve and gathering input from stakeholders such as radiologists and hospital leaders to ensure smooth integration [38]. More real-world studies and clinical trials are required to close this gap [38]. Additionally, AI developers should collaborate with radiologists to test AI tools across diverse settings to ensure they can be used safely and effectively in radiology [14].

The legal implications of AI in radiology represent an inadequately examined area that requires greater scrutiny. One of the main concerns is the question of liability when errors occur: it remains unclear whether responsibility lies with the radiologist, the AI software developer, or the hospital implementing the system [39]. This uncertainty arises because current laws are not designed to address AI-related errors and do not clearly define liability, resulting in ambiguity in cases involving AI errors [15]. Since diagnostic accuracy is critical in radiology, the appropriate governing bodies must regularly review and update regulations to ensure that AI integration enhances patient care while protecting physicians from unwarranted legal risks [40].

AI in radiology is rapidly transforming the field, making education for radiologists essential to improving the quality of care. Many radiology trainees have expressed concern that they feel underprepared due to limited exposure to AI education, which hinders their ability to critically evaluate and apply AI tools effectively [37]. Without proper training, radiologists may overlook AI’s limitations, misinterpret outputs, or fail to identify potential biases [41]. For example, Zeng et al. examined the errors an AI algorithm made when reading mammograms and found that false positives and false negatives were the most common issues [42]. Such findings highlight the need for education and training programs that help radiologists understand how AI models function, interpret results appropriately, and assess their performance [41]. In addition, implementing AI education can help hospitals enhance the safe integration of these technologies, reduce the risk of errors, and increase radiologists’ confidence in using AI as a decision-making tool [37].

Methodological considerations and limitations

A search on PubMed was conducted using specific filters (Clinical Study, Journal Article, Full Text; Species: Humans; Language: English; Publication dates: 2017-2025) and applied the NICE guidelines, which may have excluded relevant studies from other databases. Only articles in English were reviewed, which may have introduced language bias. The inclusion criteria focused on articles related to AI in radiology and AI in fracture detection, specifically clinical studies, systematic reviews, meta-analyses, and technical papers directly relevant to these subjects. The review concentrated on studies published from 2017 to 2025, with most references originating from recent years, making it difficult to determine the completeness of the recent literature captured. Case reports, editorials, and non-human studies were excluded, potentially omitting important context or novel experimental research. PubMed was searched using the following terms: (“artificial intelligence” OR “AI” OR “machine learning” OR “deep learning” OR “convolutional neural networks”) AND (“fracture detection” OR “radiology” OR “AI in fracture detection” OR “AI in radiology”).

The studies included varied in sample size, study design, and AI model training datasets, which could affect generalizability and introduce potential biases. This review also provides examples of AI-assisted fracture detection; however, it does not include pooled quantitative data, such as sensitivity and specificity, across all fracture types. Lastly, since this is a narrative review, a meta-analytic synthesis was not performed; therefore, the evidence presented remains descriptive and not quantitatively analyzed. These limitations highlight the need for continuous and systematic reviews to evaluate AI applications in fracture detection more thoroughly.

## Conclusions

AI is rapidly transforming the field of radiology by enabling more accurate fracture detection and diagnosis on imaging while also optimizing workflows. This AI capability has improved the quality of care and enhanced hospital efficiency by enabling more efficient care delivery. AI has also shown that it can speed up the process of scan reporting, especially in emergencies. For instance, in trauma cases, AI can help detect complex fractures that doctors might miss, speeding up treatment decisions and improving patient outcomes.

The incorporation of AI into radiology poses numerous challenges that warrant further investigation. Algorithmic bias, threats to professional autonomy, and the safety of patient data are some of the most important ethical issues. At the same time, clinical translation and ambiguous legal liability persist as substantial obstacles to the safe deployment of these technologies. To solve these problems, a long-term governance framework, active stakeholder involvement, and real-world testing of AI tools are required. Furthermore, it is important to include AI education in radiology training programs so that trainees can critically assess and use AI in clinical practice. Getting patients involved in the process can make the healthcare system even more transparent and reliable. In conclusion, responsibly utilizing the advantages of AI in radiology while addressing these gaps can enhance patient outcomes.
